# Reduction of *Ascaridia galli* Pathology by *Salmonella typhimurium* in Broiler Chicken

**DOI:** 10.1155/2021/5386575

**Published:** 2021-09-04

**Authors:** Eric Igor Sop Foka, Cedric Yamssi, Ben Enyetornye, Christelle Noumedem Anangmo, Mpoame Mbida, Theodore B. Mayaka

**Affiliations:** ^1^Department of Animal Biology, Faculty of Sciences, University of Dschang, Cameroon; ^2^Department of Biomedical Sciences, Faculty of Health Sciences, University of Bamenda, Cameroon; ^3^School of Veterinary Medicine, University of Ghana, Legon, P.O. Box LG 139, Accra, Ghana; ^4^Department of Microbiology, Hematology and Immunology Faculty of Medicine and Pharmaceutical Sciences, University of Dschang, Cameroon

## Abstract

Previous studies have reported interactions between *Salmonella* spp. and some helminth coinfections. In this study, *S. typhimurium* and *Ascaridia galli* coinfections were analyzed, and the consequences on therapy were proposed. In a first experiment where the effect of the bacteria on the nematode was evaluated, thirty 42-day-old broiler chickens were divided into 3 groups and coinfected with both parasites. The rate of *A. galli* egg inoculum was kept constant at 500/ml while that of *S. typhimurium* varied as follows: T_0_ (500 *A. galli* eggs/ml), T_104_ (500 *A. galli* eggs/ml+104 *S. typhimurium* CFUs), and T_106_ (500 *A. galli* eggs/ml +10^6^*S. typhymurium* CFUs). EPG and parasitic load were measured using the McMaster technic, and number of worms and their length were also measured. We observed that T_106_ containing 10^6^ CFUs of *Salmonella* significantly reduced the EPG values, and this group recorded the lowest worm load ranging from 18 to 21 worms. Likewise, the length of the worms obtained with T_104_ and T_106_ appeared to be shorter than those of the control (T_0_). In a second experiment to assess the effects of the nematode on the bacteria, thirty 42-day-old broiler chickens were divided into 3 groups and coinfected with both parasites. The rate of *S. typhimurium* inoculum was kept constant at 10^6^ CFUs while that of *A. galli* varied as follows: T_0_ (10^6^CFUs), T_500_ (500 *A. galli* eggs/ml +10^6^*S. typhymurium* CFUs), and T_750_ (750 *A. galli* eggs/ml +10^6^*S. typhymurium* CFUs). Bacterial load was measured using Voogt technique. We observed that T_500_ increased the colonization time and prolonged the duration of *S. typhimurium* secretion. *Salmonella* appears to be a hyperparasite considering the deleterious effect on *A. galli*. Due to this, it may be prudent to combine anti-*Salmonella* treatment with anthelmintic so as to effectively treat pathologies caused by these two pathogens.

## 1. Introduction

The study of the interaction between Gram-negative bacteria and parasites dates back to decades where [1] suggested *Strongyloides* can drag *Escherichia coli* during their migration from the intestine to other organs of the host. Other nematodes such as *Trichinella spiralis* and *A. galli* have been identified as direct vectors for *Salmonella* transmission [2, 3]. Laboratory studies on schistosomes showed that *Salmonella* can colonize the intestinal tract of *Schistosoma mansoni* [4]. [5] demonstrated the association of *Salmonella* and schistosomes by culturing *Salmonella paratyphi* from certain schistosomes. Several experimental studies have begun to demonstrate the persistence and enhanced growth of *Salmonella* in chickens concurrently infected with *Ascaridia galli* [6]. In this regard, the presence of *A*. *galli* worms *in vivo* indicates that chickens were infected transiently with *Salmonella typhimurium.* The identification of *Salmonella* organisms within the gut or on the tegument of *A. galli* worms further indicated an intimate association between these two pathogens [7]. This study provides a baseline for the understanding of the frequently noted clinical signs associated with chronic salmonellosis in chickens [8].

Several studies have shown the major importance of the association between these bacteria (*S. typhimurium*) and the helminth (*A. galli*). These studies focused not only on the pathogenic effects on the host but also the consequences in terms of therapy [9] as these pathogens represent a real problem for both avian and human health. This led to integration of measures complying with the One Health (OH) approach of disease prevention and control. In order to ensure food security and safety, the tripartite alliance of WHO-FAO and OIE emphasised the need to curb the spread of these pathogens between and among animal and humans [10]. The threat of these pathogens is that they cause zoonoses which is linked to the consumption of poultry meat or products. Research findings of [6] revealed that *A. galli* could play a major role in the transmission of *Salmonella* infections, consequently increasing the economic losses in the agro-pastoral sectors. The total cost losses are estimated to be between 15.5 million USD to 2.8 billion dollars yearly in the United States of America [11].

The clinical manifestation of salmonellosis alone seems to differ significantly from situations of coinfection. In the case of schistosomes [12] and *A. galli* [13], the treatment of salmonellosis becomes difficult or even impossible when it is associated with schistosomes [9]. The transmission of the bacteria can be done by *A. galli* eggs [13] indicating a possible interaction between them. However, there is paucity of information on these interactions especially one involving *A. galli*. A better understanding of these interactions could help explain the pathologies caused by these two parasites. This study was undertaken to explore the interactions that could exist between *Salmonella typhimurium* and *A. galli* as well as predict the implications for therapy.

## 2. Material and Methods

### 2.1. Study Location

The study was conducted in the Biology and Applied Ecology laboratory and in the Physiology and Animal Health laboratory of the University of Dschang, Cameroon.

Experimental animals: The broiler chickens (*Abor acres*) breed used in this study were obtained from commercial poultry supplier (Cameroon Provender Company) based in Dschang, Cameroon.

### 2.2. Source of *S. typhimurium*

Salmonella sample were supplied by Professor LoVerde Philip of the University of Texas Health, USA.

### 2.3. Source of *A. galli* Eggs and Infection

Local chickens naturally infected with *A. galli* were humanely euthanized. Adult worms were recovered from their intestines and rinsed twice in phosphate-buffered saline (PBS, pH: 7.2). Their eggs were removed from worms' uteri and then incubated for 21 days in 0.1 N sulfuric acid to obtain embryonated eggs [14]. After egg embryonation, chickens previously raised up to 42 days were infected with *A. galli* eggs. Chickens were sacrificed 30 days postinfection, and the adult worms were removed from the intestines.

### 2.4. Experiment 1: Effects of *S. typhimurium* on *A. galli* Coinfection on the Hosts

For the evaluation of the bacteria effect on the nematode, thirty 42-day-old “Arbor acres” breed of broiler chickens was divided into 3 equal groups as described by [6] but with little modification and coinfected with both parasites. The rate of *A. galli* egg inoculum was kept constant at 500/ml while that of *S. typhimurium* varied as follows: T_0_, T_104_, and T_106_ (Table 1). On the 30^th^ day postinoculation, parameters such as EPG, parasitic load, and length of worms were measured.

### 2.5. Concentration of Fecal Egg (EPG)

To determine the EPG, stool samples were taken on daily basis in all experimental groups for 21 days. The samples were stored in 10% formalin and then examined qualitatively (flotation technique) to confirm infection and quantitatively (Mc Master technique) to determine the EPG. Two grams (2 g) of stool samples were mixed with 60 ml of saturated saline solution (400 g of NaCl in 1liter of distilled water). Since the stool samples were preserved in formalin, it was difficult to extract 2 g of stool from the mixture, so the total weight (MT) of every flask containing stool sample, the weight of 10 ml of formalin (*m*_1_), and the empty flask weight (*mt*) were measured, respectively. The exact stool weight (*mmf*) within each flask was then determined by the following formula: mmf = MT–(*m*_1_ + mt).

The proportion of stool percentage within every flask (% mmf) was obtained by using the formula %mmf = mmf × 100/(mmf + *m*_1_).

To deduct of the stool-formalin mixture (*Yg*) and to determine *Xg* with the formula Xg = %mmf × Yg, *Xg* was determined using Willis and Mc Master techniques.

### 2.6. Parasitic Load and Measurement of Parasite Length

Chickens were euthanized 52 days postinfection. Adult worms and larvae were then extracted from the intestine. After extraction of the worms, females were separated from the males by observing the posterior parts using a Pierron® brand binocular loop. The length of each worm was measured using a graduated ruler to identify stunted worms.

### 2.7. Experiment 2: Effects of *A. galli* on *S. typhimurium* Coinfection on the Hosts

For the evaluation of the nematode effect on *Salmonella typhimurium*, thirty 42-day-old “Arbor acres” breed of broiler chickens was also divided into 3 equal groups and coinfected with both parasites. The rate of *S. typhimurium* inoculum was kept constant at 10^6^ CFUs while that of *A. galli* varied as follows: T_0_ (10^6^ CFUs), T_500_, and T_750_ (Table 2). The bacterial load was monitored at 3 days interval for 21 days postinfection. Cloacal swabs were taken to measure bacterial load as described by [15]. The solutions obtained were incubated on *Salmonella*-*Shigella* agar at 37°C for 24 to 48 h to enumerate the *Salmonella* colonies.

### 2.8. Statistical Data Analysis

Statistical analyses were done using the R software, version 3.5.3 of [16], mainly for categorical analyzes [17], in R. To gain a better understanding of the effects of *Salmonella* on the nematode, the frequencies of reported EPG means were tested by Satterthwaite's *t*-test, while those of the length of *A. galli* worms were assessed through the Shapiro-Wilk Normality Test. Furthermore, the effects of nematode on bacteria were examined by applying nonparametric polynomial regression to differentiate the means of the logarithm of the bacterial load. All tests were performed at 5% probability level.

## 3. Results

### 3.1. Experiment 1: Effects of *S. typhimurium* on *A. galli* Coinfection on the Hosts

**Figure 1:**
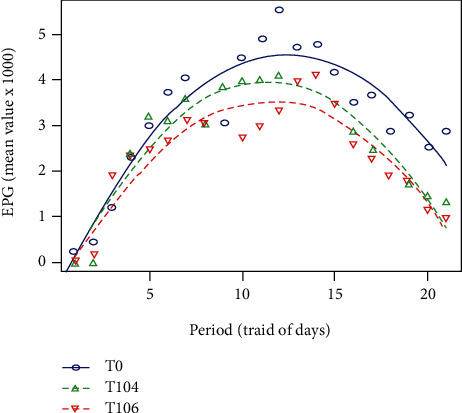
*Ascaridia galli* fecal eggs (EPG) according to *Salmonella typhimurium* concentrations and time. T_0_: concentration 500 *A. galli* ova +0CFUs of *Salmonella typhimurium*; T_104_: 500 *A. galli* ova +10^4^ CFUs *Salmonella typhimurium*; T_106_:500 *A. galli* ova +10^6^ CFUs of *Salmonella typhimurium*.

Figure 1 shows variations in EPG in the three treatments. The raw data on the EPGs were transformed to produce normally distributed data. Positive fecal egg counts were detected; the first-time individual samples were taken. EPGs varied over time with a bell-shaped structure and a peak occurring around the 12^th^ day. There is also a variation in EPGs based on the treatments. The lowest values were obtained with T_106_ while the highest values were recorded with T_0_. In addition, the duration of *A. galli* excretion with the same treatment was relatively shorter than those of the other groups. T_106_ containing 10^6^ CFUs of *Salmonella* significantly reduced the EPG values. T_104_ produced intermediate effects in comparison with the control and T_106_.

The parabolic regression analysis showed that the intercept, the linear, and the quadratic terms were highly significant over time. Thus, EPGs of the three concentrations of *Salmonella typhimurium* differ significantly (*P* < 0.05) from each other. The significant differences between the concentrations of *Salmonella typhimurium* can clearly be observed from the following parabolic regression equations:

(1) $$\begin{array}{c} \left[{\text{T}}_{0}\right]\beta_{0}:-0.624+0.835t-0.0335t^{2},\\ \left[{\text{T}}_{104}\right]\beta_{1}:-0.624+0.809t-0.035t^{2},\\ \left[{\text{T}}_{106}\right]\beta_{1}:-0.624+0.726–0.0315t^{2}. \end{array}$$

### 3.2. Effect of *Salmonella typhimurium* on Parasitic Load

**Figure 2:**
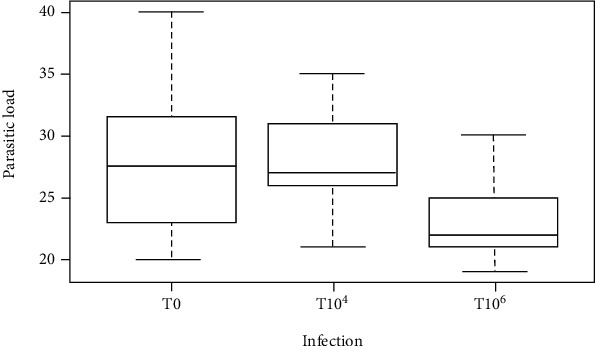
*Ascaridia galli* parasitic load in infective host groups with varying Salmonella *typhimurium* concentration. T_0_: concentration 500 of *A. galli*+0CFUs *Salmonella typhimurium*; T_104_: concentration 500 *A. galli*+104 CFUs *Salmonella typhimurium*; T_106_: concentration 500 *A. galli*+106 CFUs de *Salmonella typhimurium*.

Occasionally, expelled worms have been found in the feces of the birds during the experimental period. The number of worms recorded at postmortem at the end of the experiments is shown in the boxplots (Figure 2). There was great variability in the number of worms per host in the different groups. No other gastrointestinal parasites except *A. galli* were seen at postmortem. The distribution of worms in each boxplot is divided into four equal areas (25%). From these boxplots (Figure 2), the median was 28 worms in T_0_. In the same group, birds with lowest worm load were recorded between 20 and 23 worms while the highest worm load was between 32 and 40 worms. T_106_ group recorded the lowest worm load ranging from 18 to 21 worms. At 10^4^ CFUs, worm's median was 27 worms, and the lowest worm load was between 28 and 35 worms. Analysis of the general linear model showed that the parasitic load obtained with T_0_ (Table 2) significantly differed from the other concentrations (*P* < 0.001). There was no significant difference between the parasitic loads obtained with double parasitism (T_104_ and T_106_) although 10^6^ CFUs (Table 2) of *S. typhimurium* reduced the number of worms (larvae + adults) considerably. The effect of monoparasitism was not to double parasitism (*P* < 0.05).

### 3.3. Effect of *Salmonella typhimurium* on the Length of *Ascaridia galli* Worms

**Figure 3:**
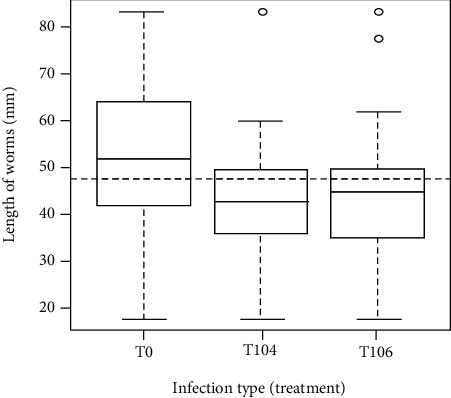
Effect of *Salmonella typhimurium* on *Ascaridia galli* worm's length. T_0_: concentration 500 worm ova *A. galli*+0CFUs *Salmonella typhimurium*; T_104_: concentration 500 *A. galli*+104 CFUs *Salmonella typhimurium*; T_106_: concentration 500 *A. galli*+106 CFUs *Salmonella typhimurium*.

The variation in length of *A. galli* as a function of treatments is presented in Figure 3. From this figure, the median of the length of the worms was 52 mm in the control group (T_0_). In the same group, the medium-sized worms recorded were between 42 and 63 mm while the highest size worm was between 63 and 83 mm worms. In T_104_ and T_106_, the medians were 42 and 45 mm, respectively. The maximum length of worms in these groups (T_104_ and T_106_) were 60 and 62 mm, respectively. The Shapiro-Wilk normality test showed that there was a gender effect on worms' length. It was observed from this test that male worms were significantly shorter than female worms (*P* = 0.005). There was a significant (0.05) length reduction in worms from birds in T_106_ group compared with the controls. There was no significant (*P* > 0.05) difference between the interaction of treatments on sex. Likewise, the length of the worms obtained with T_104_ appeared to be shorter than those of the control, but not statistically significantly (*P* = 0.465). However, the length obtained with the T_106_ was significantly shorter than those of the control (*P* = 0.01).

### 3.4. Experiment 2: Effects of *A. galli* on *S. typhimurium* Coinfection on the Hosts

**Figure 4:**
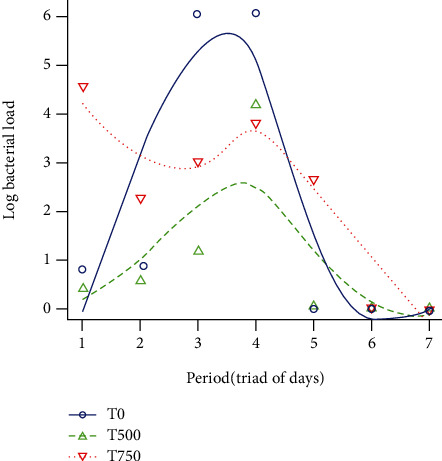
Bacterial load after oral inoculation of broiler chicken with *Salmonella typhimurium*. T_0_: concentration 10^6^ CFUs de *Salmonella typhimurium* +0 *A. galli*; T_500_: concentration 10^6^ CFUs de *Salmonella typhimurium* +500 *A. galli*; T_750_: concentration 10^6^ CFUs de *Salmonella typhimurium* +750 *A. galli*.

The variation of the bacterial load over time is shown in Figure 4. The bacterial load varied depending on the concentration of the inoculum. We have observed two bell curves (T_0_ and T_500_) and an inverted sigmoid (T_750_). The highest bacterial load was recorded with the T_0_ (Table 2) inoculum, with a maximum value occurring on the 11^th^ day. However, the duration of excretion of the bacteria in T_0_ remained relatively short. With the T_750_ inoculum, the bacterial load was less but with a prolonged duration of secretion as seen in Figure 4. The bacterial load at the T_500_ concentration was slightly higher than the one obtained with T_750_. The analysis of the nonparametric polynomial regression showed that the values of the bacterial load obtained with the inoculum T_0_ differed significantly from the other concentrations (*P* < 0.001). Moreover, the bacterial load varied significantly from one group to another (*P* < 0.05). Thus, *A. galli* prolonged the duration of *Salmonella typhimurium* secretion.

## 4. Discussion

Considering that *A. galli* egg excretion was higher in the control group than those in the coinfected group, it suggests that the best method for the detection of *A*. *galli* perhaps is the manual count of eggs in the stool. Only mature female worms laid eggs, and the diagnosis is only possible after a heavy infection. In addition, *Salmonella* infected reproductive tissues and cause ovarian and testicular abscesses, which affects egg formation and drastically reduces parasite's egg-laying rate [18]. Testicular abscesses cause the inhibition of ascaroside secretion by male worms. Nematodes secrete ascarosides as pheromones to induce egg formation as well as to control various behavior including female attraction to males and adult aggregation [19, 20].

Parasites generally show high reproductive potential which is responsible for exponential growth in numbers, which is a characteristic of microorganisms. Egg production is often high when we consider the daily laying rate and the parasite longevity. In the case of *Ascaris*, it lays about 200,000 eggs per day, with a life span of nine months [21]. It may therefore be reasonable to expect a higher *A. galli* egg excretion in the coinfected group, since these two parasites occupy the same niche within hen's intestine [22]. This study demonstrated that *S. typhimurium* may play an important role in reducing the egg laying ability of *A. galli*, as the values of EPG obtained in coinfected groups were lower than those in the control. This could be explained by the ability of the bacteria to infect reproductive tissues like the oviduct and the ovaries. These tissues serve as a gate way for the contamination of eggs in hens [18, 23].

It is generally accepted that the establishment of *A. galli* in chicken intestine is influenced by many factors such as age, infectious dose, sex, and the host diet. A report by [4] using various bacterial species found that certain species of bacteria, including *Klebsiella* sp. and *Escherichia coli*, rapidly colonize *Schistosoma mansoni* and subsequently lead to the death of the worms. These authors further indicated that *S. typhimurium* naturally present in mice infected with *S. mansoni* causes the death of all the worms. The length of the worms extracted inside the control group was bigger in size than those extracted from the coinfected groups. It is possible that the expulsion of the worms had some impacts on the average load of the worms. This difference in size and parasitic load observed can be a consequence of competition for space and nutrients because the two pathogens (*Salmonella* and *A. galli*) occupy the same site which is the intestine. These pathogens must also find a balance in order to keep the host alive, as death of the host will eventually lead to parasite's death. Male worms outnumbered female worms in all the experimental groups. This phenomenon allows each adult female worm to have at least one male for mating in order to lay fertile eggs, to ensure the survival of the species [21]. The number of eggs that a female worm can lay also explains the fecal egg concentration obtained in this study. The likelihood of *A. galli* establishing infection has been shown to be significantly higher in chickens [6].

The specific effects of *Salmonella* on the integrity and viability of worms have not been reported in other studies. Based on the findings from this study, it is obvious that *Salmonella* has variable effects on *A. galli* including the reduction of growth, laying rate, and death of worms. The average bacterial load follows the Gaussian curve. The decrease of the bacterial load in coinfected groups is due to interspecific competition. The value of the bacterial load was greater in the control group, but the duration of excretion of these bacteria was longer in the coinfected groups. *Salmonella* excretion is believed to be favored by *A. galli* when *S. typhimurium* is found in the digestive tract and uterus of *A. galli* [7] Competing infections with *A. galli* and other bacteria have shown that *A. galli* facilitates bacterial infections [23] This may be related to the polarization of the immune response; the immune response of mammals is known to polarize in type 1 (Th1) or type 2 (Th2) immune pathways depending on the type of pathogen encountered [24]. Similar findings have been reported by [25] in chickens. Thus, helminth infection could suppress the Th1 response and indirectly favor the establishment of bacterial infection and vice versa. Dendritic cells stimulated by helminth infections play an important role in the regulation of the intestinal immune response as well as in the modulation of bacterial pathogenesis as described in [26] This is because helminth infections alter host's response to competing bacterial infections and promote bacterial intestinal binding via a novel mechanism that requires activation of dendritic cells and expression of interleukins-10 (IL-10) [26].

These results further provide plausible reasons for the difficulty of antimicrobial therapy in both natural infections with *Salmonella* when there is coinfection with *Ascaridia* spp. in chicken and even in experimental models. Some researcher suggested that the treatment of salmonellosis can be achieved by simply deworming the host, thus suggesting the dependence of the bacteria on the presence of live worms *in vivo* [27]. Moreover, other researchers reported that parasitic helminths are linked to a number of changes in host's organs, which facilitate *Salmonella* infections [28, 29]. However, the potential variability of host cell interactions continues to complicate the understanding of the response of *A. gallus* to *S. typhimurium* infections [8]. [29] compared five serovars of *Salmonella* that infect poultry (*S. typhimurium*, *S. enteritidis*, *S. Heidelberg*, *S. Kentucky*, and *S. Senftenberg*) so as to assess cell invasion by *Salmonella*, its intracellular survival mechanisms, modulation of phorbol myristate acetate (PMA), and burst oxidative activity of nitric oxide in a chicken macrophage cell line transformed by the MC29 virus and HD-11 were evaluated. *Salmonella enteritidis* and *S. typhimurium* did not generate any detectable production of nitric oxide [29]. In addition, the complexity of the interaction between the microflora of the gastrointestinal tract, the immune system, and host-dependent factors such as age, stress level, and general health may play an important role in host-pathogen interactions and the reduction of *Salmonella* excretion [30, 31] The increased susceptibility to entero-pathogens of bacterial origin by helminth infections could contribute to failure of the use of *Salmonella* vaccines in countries with high helminth prevalence, since helminths modulate the immune response against *Salmonella*-based vaccines [32].

## 5. Conclusion

*Salmonella typhimurium* has deleterious effects on *A. galli* by reducing the nematode length and load in broiler chickens. This is in agreement with other studies that demonstrated that *Salmonella* interacts with gastrointestinal nematodes when they live together in a common environment. Moreover, the effects of this interaction between *S. typhimurium* and *A. galli* have been shown to be harmful both for the *A. galli*. This is manifested in the reduction in pathogenicity of *A. galli*. In view of these bacteria, deleterious effect on *A. galli* and *Salmonella* appears to be a hyperparasite. It would be wise to recommend the association of anti-*Salmonella* treatment with anthelmintic for the treatment of pathologies caused by these two pathogens. This needs to be considered in the future to augment other control strategies in poultry.

## Figures and Tables

**Table 1:** Experimental design-1.

No.	Group size	Experimental groups	*A. galli* inoculum eggs	*Salmonella typhimurium*/CFUs
T_0_	10	*A. galli*	500	0
T_104_	10	*A. galli + Salmonella typhimurium*	500	10^4^
T_106_	10	*A. galli + Salmonella typhimurium*	500	10^6^

**Table 2:** Experimental design-2.

No.	Group size	Experimental groups	*A.galli* inoculum eggs/ml	*Salmonella typhimurium* CFUs
T_0_	10	*S. typhimurium*	0	10^6^
T_500_	10	*A. galli + S. typhimurium*	500	10^6^
T_750_	10	*A. galli + S. typhimurium*	750	10^6^

## Data Availability

The datasets generated and analysed in the current study can be made available by the corresponding author upon reasonable request.
